# Supplementary material for: Disentangling edge and bulk spin-to-charge interconversion in MoS2 monolayer flakes
Source: Nat Commun. 2025 Mar 30;16:3075. doi: 10.1038/s41467-025-58119-4 (PMC11955567; doi:10.1038/s41467-025-58119-4)
Supplement: Supplementary file 1 — Supplemental Information [file 41467_2025_58119_MOESM1_ESM.pdf]

Supplementary Information for

Disentangling edge and bulk spin-to-charge interconversion in MoS_2 monolayer flakes

Rodrigo Torrão Victor[1], Syed Hamza Safeer[1,2], John F. R. Marroquin[3], Marcio Costa[4], Jorlandio F. Felix[3], Victor Carozo[5], Luiz C. Sampaio[1], and Flávio Garcia[1,*]

[1] Centro Brasileiro de Pesquisas Físicas, R. Dr. Xavier Sigaud, 150 Urca, Rio de Janeiro RJ, 22290-180, Brazil.

[2] Materials Science Laboratory, Department of Physics, Quaid-i-Azam University, Islamabad, 45320, Pakistan.

[3] Instituto de Física, Universidade de Brasília, Brasília, DF, 70830-200, Brazil

[4]Instituto de Física, Universidade Federal Fluminense, Niterói, RJ 24210-346, Brasil.

[5]Departamento de Física, Pontifícia Universidade Católica do Rio de Janeiro, Rio de Janeiro, RJ 22451-900, Brasil.

[*]To whom correspondence should be addressed; E-mail: fgarcia@cbpf.br

S1 MoS_2 flake quality

To support the quality of the MoS_2 flakes and the transfer method, several measurements were made in the as-growth flakes before transfer ($Si/SiO_2/MoS_2$) and after transfer ($GGG/YIG/MoS_2$). These measurements are addressed in this section.

S1.1 Size control

As discussed in the manuscript, by keeping the growth conditions and increasing the growth time, triangular MoS_2 flakes continue to grow and eventually coalesces to form a uniform polycrystalline MoS_2 film, still as monolayer [1, 2]. This behaviour can be seen in images obtained by optical microscopy (see Figs. S1(a-d)), which shows MoS_2 flakes at different growth times.

S1.2 Optical microscopy before and after the MoS_2 transfer

To demonstrate the quality of the etch-free MoS_2 flake transfer method [3], Fig. S2(a) shows an optical image of a typical sample ($Si/SiO_2/MoS_2$). As can be seen, the Ambient Pressure Chemical Vapor Deposition (APCVD) process results in MoS_2 crystals shaped as equilateral triangles. A sulfur-rich atmosphere favors the growth of MoS_2 with sulfur atoms at the ends, resulting in crystals with H symmetry and zigzag edges. This symmetry is detailed in Fig. S2(a), where the blue spheres represent molybdenum and the orange spheres represent sulfur. As can be seen from Fig. S2(b), the shape and morphology of the MoS_2 monolayers are preserved when transferred to the YIG ($GGG/YIG/MoS_2$), indicating that the transfer occurred without damage to the flakes.

S1.3 Raman spectra

To further support the quality of the flakes, Raman spectra are shown in Fig. S3(a), for both the as-grown (red) and the transferred MoS_2 (black). As can be seen, the in-plane phonon mode E^1_{2g} at 379.4 cm^{-1} and the out-of-plane mode A_{1g} at 399.5 cm^{-1} remained unchanged. The difference between the peak positions was 20.1 cm^{-1}, corresponding to a monolayer [4]. This result shows that the crystals were not damaged during the transfer process

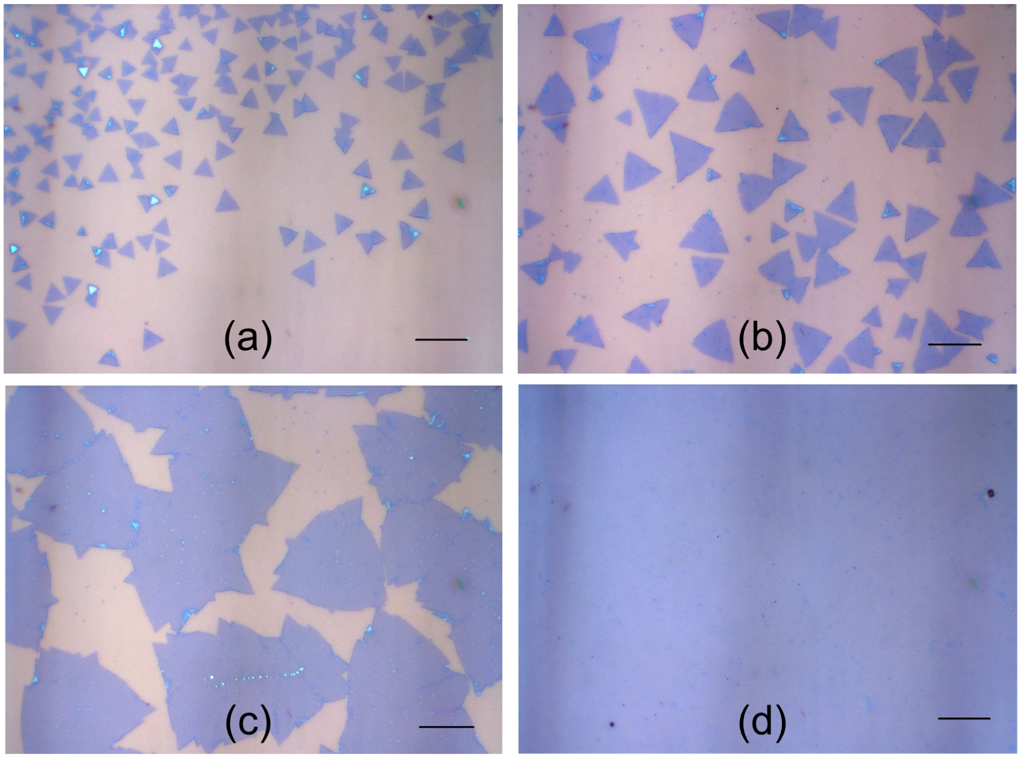

Figure S1: (a-d) Images obtained by optical microscopy showing the growth of the triangular MoS_2 monolayer flakes up to a large-area filling the film. The scale bar is 20 μm.

and did not show significant changes in lattice parameters and roughness. Fig. S3(b) shows the Raman spectrum of GGG/YIG, both before (blue) and after (red) the transfer of MoS_2 for a wider range. In the post-transfer spectrum, the in-plane and out-of-plane modes A_{1g} and E^1_{2g} of MoS_2 are identified, along with the other peaks previously measured before the transfer and attributed to the YIG and GGG modes. No other peaks were identified, confirming that no contamination or defects were found after the transfer process. It is important to note that our samples show a red shift of about 6 cm^{-1} compared to exfoliated MoS_2 samples. As the Raman calibration was performed using the substrate's silicon peak at 520 cm^{-1}, this red shift is probably due to strain between the sample and the substrate surface. Tensile strain occurs as a result of substrate interaction or synthesis conditions that can cause a redshift in Raman peaks. This is caused by stretching the lattice,

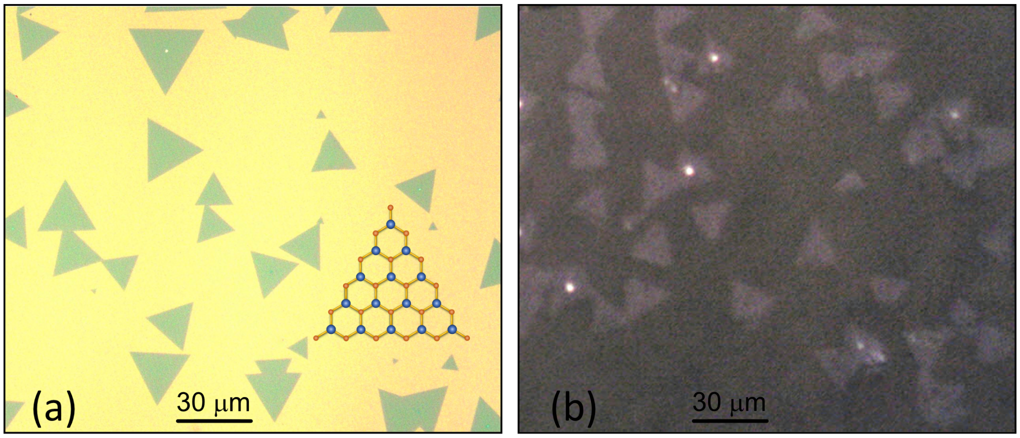

Figure S2: Optical images of the triangular MoS_2 monolayer in (a) Si/SiO_2 and (b) GGG/YIG. The scale bar is 30 μm.

which reduces the phonon frequencies. CVD grown samples can have residual stresses due to growth conditions (temperature / pressure) [2].

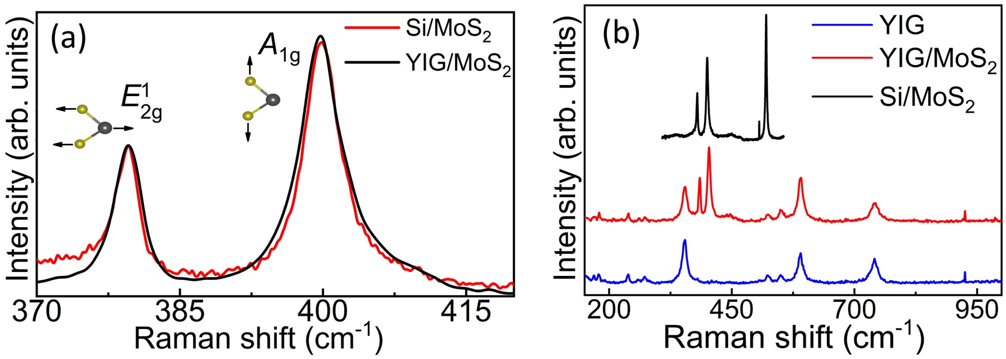

Figure S3: Normalized Raman spectra of MoS_2 flakes. In (a), GGG/YIG/MoS_2 in black and Si/SiO_2/MoS_2 in red. In (b), GGG/YIG in blue, GGG/YIG/MoS_2 in red, and Si/SiO_2/MoS_2 in black.

S1.4 Raman map

Optical microscopy showed a very low contrast for the samples transferred to the YIG compared to images performed on samples with silicon as substrate

(see Fig. S1). Raman mapping was therefore performed in some regions of YIG/MoS_2. Fig. S4 shows a map of the intensity of the A_{1g} mode peaks for one of these crystals. Like all the measured flakes, this representative map shows a uniform intensity distribution within the triangular flakes. At the edges, the intensity is slightly lower due to the characteristics of the beam used for mapping. This map highlights the uniformity and structural quality of the crystals, as well as their preservation after transfer.

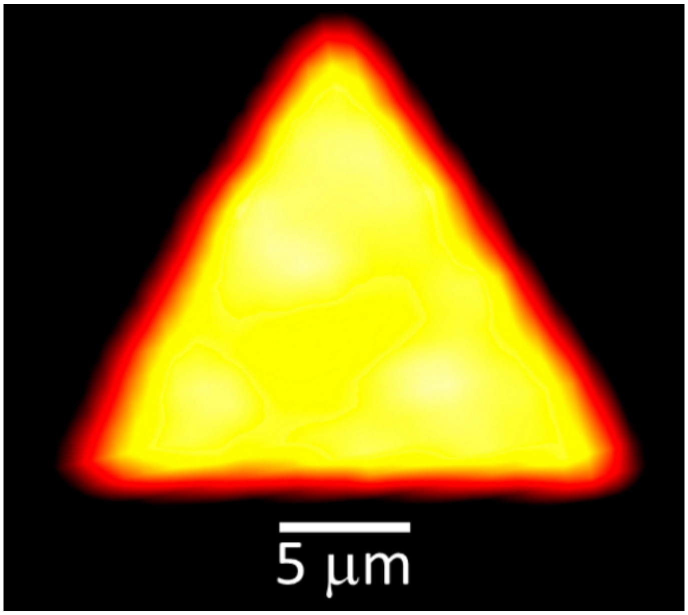

Figure S4: Raman mapping of a MoS_2 flake obtained from the intensity of the A_{1g} mode. The scale bar is 5 μm.

S1.5 Photoluminescence spectroscopy

The photoluminescence spectrum was recorded before and after transferring the MoS_2 flakes. In both scenarios, the observation was limited to a single neutral exciton peak located at 1.83 eV (670 nm), as shown in Fig. S5. A single and intense peak in the photoluminescence spectrum attests to the high optical quality of the MoS_2 flakes. This intense photoluminescence is associated with a direct band gap, which supports the conclusion that it is a monolayer of MoS_2, since this material in volumetric form or bilayers presents an indirect band gap.

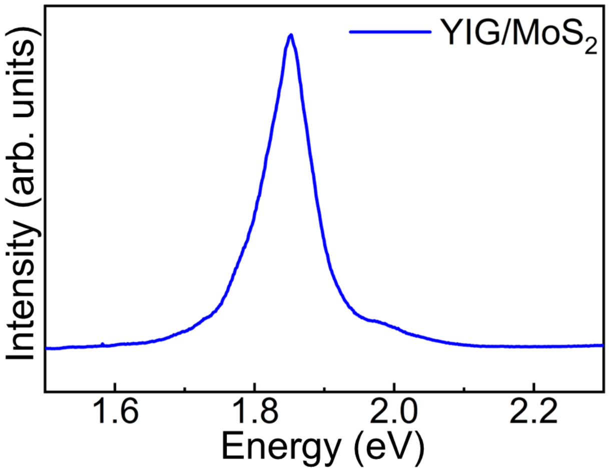

Figure S5: A photoluminescence spectrum of representative of YIG/MoS_2 heteroestructure.

S1.6 Atomic force microscopy

To further support that the flakes are monolayers, atomic force microscopy was performed in several flakes, in different samples. As can be seen in Fig. S6 for a typical flake, the difference in height of the flakes is 0.9 nm, corresponding to a MoS_2 monolayer.

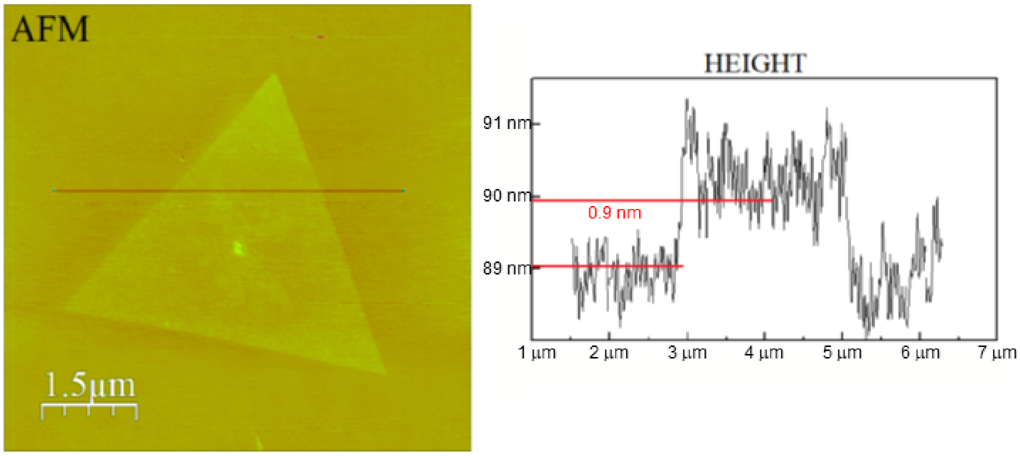

Figure S6: AFM image of a MoS_2 triangular flake. The line profile shows a height of 0.9 nm, corresponding to an atomic monolayer.

S1.7 Light absorption

One of the key aspects of this work is the light-driven spin pumping. To corroborate the discussion on the light absorption of MoS_2, Figure S7 shows the light absorption of MoS_2 as a function of the incident light wavelength in a broadband. As can be seen, there are several resonance absorption peaks. It is important to highlight the presence of a very intense peak in the range of 400 to 440 nm, which encompasses the 405 nm violet laser used to obtain the light-driven spin pumping.

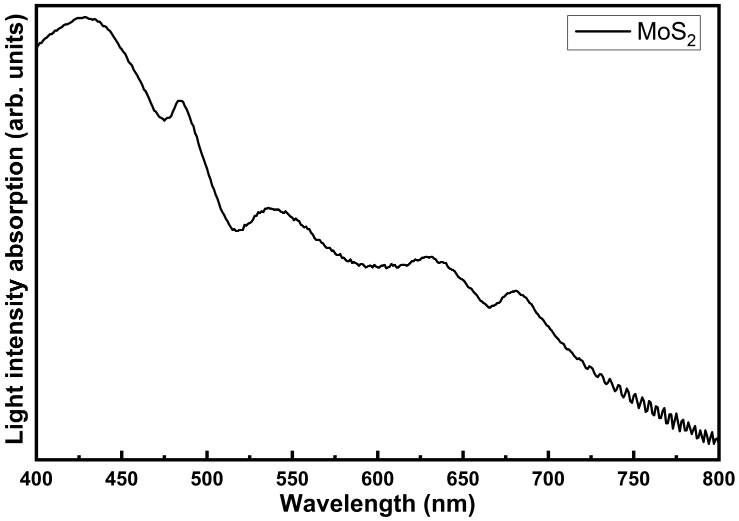

Figure S7: MoS_2 light absorption intensity as a function of incident light wavelength.

S1.8 Samples appearance

One of the most important results discussed in the manuscript is related to the aspect ratio of the MoS_2 flakes covering the YIG film. It is therefore important to present some features of each sample used. To facilitate the sample identification, Figure S8 shows the spin pumping as a function of the area/perimeter ratio for MoS_2 flakes, similar to Figure 1e (see manuscript). As each point represented by blue circles corresponds to a given sample, they were labeled from *(i)* to *(xiii)*.

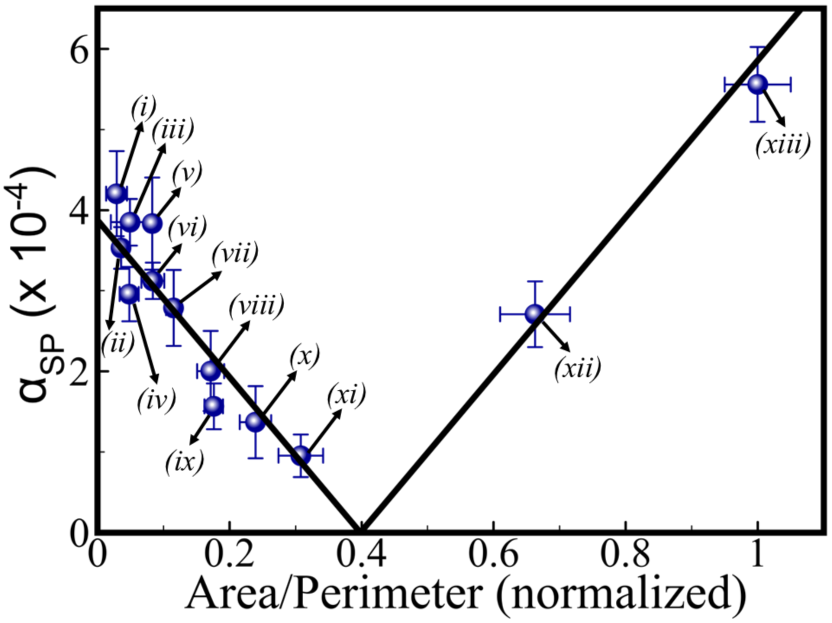

Figure S8: Spin pumping (α_{SP}) dependence as a function of the total MoS_2 area divided by the total perimeter (A_{Total}/P_{Total}). Each point corresponds to a sample numbered from left to right from *(i)* to *(xiii)*. The uncertainty in α_{SP} was obtained from the data fit and in area and perimeter from the image analysis.

Figures S9 and S10 show images of the whole set of samples used. The images were obtained using a Zeiss Axio scope A1 microscope coupled to an objective lens with a magnification of 20, 50, and 100 times. All images are 1x1 mm^2 large. Since the contrast between the MoS_2 and the YIG/GGG is weak and sometimes difficult to visualize, different filters were used to obtain good images yielding different background colors.

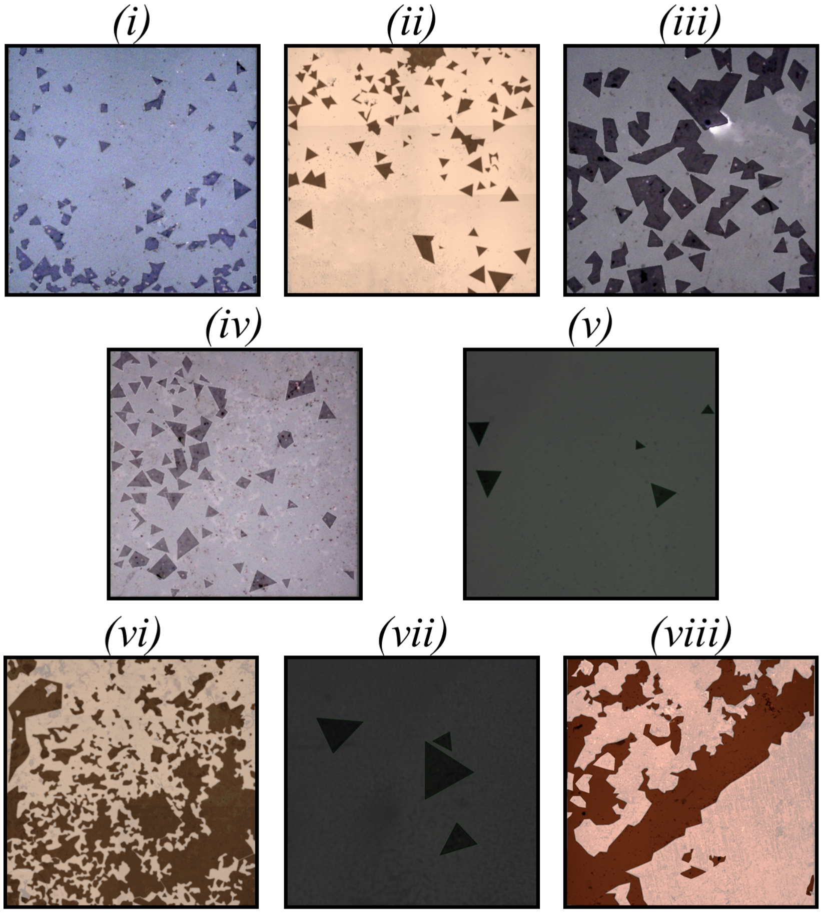

Figure S9: Optical microscopy of samples from *(i)* to *(viii)*. All images are 1x1 mm^2 large.

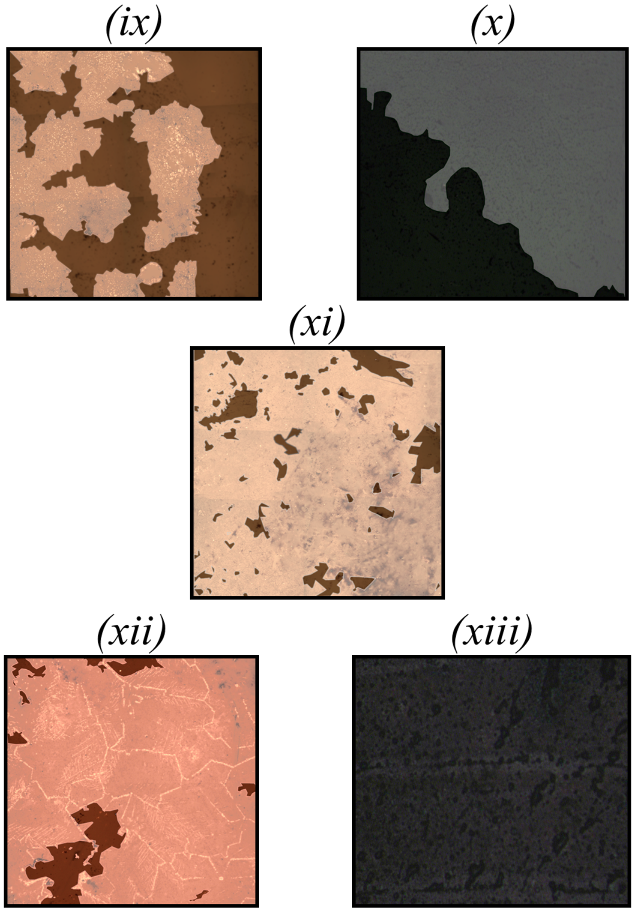

Figure S10: Optical microscopy of samples *(ix)* to *(xiii)*. All images have 1x1 mm^2.

S1.9 Edge and area quantification

The total perimeter and area of each sample was carefully evaluated by mapping and measuring the edge of all flakes in the sample in direct contact with the YIG, as shown in Figures S9 and S10. Since the optical contrast of the flakes is low, the mapping was performed by optical microscopy using different objectives and filters to enhance the contrast of the flakes with respect to the substrate. After mapping, the edge length (area) of the flakes was estimated using ImageJ software [5], and the total edge length (area) was obtained by summing up all flakes in the sample. It is important to note

that the merged edges were not used to obtain the total edge length. This means that the region where two (or more) different flakes merge to form a larger flake is not included in the quantification of the total edge length. In Figure S11 are presented three samples (*ii*, *iii*, and *ix*) to exemplify the edges. In the the left side are the images used in Figures S9 and S10, on the right side the edges are highlight in red.

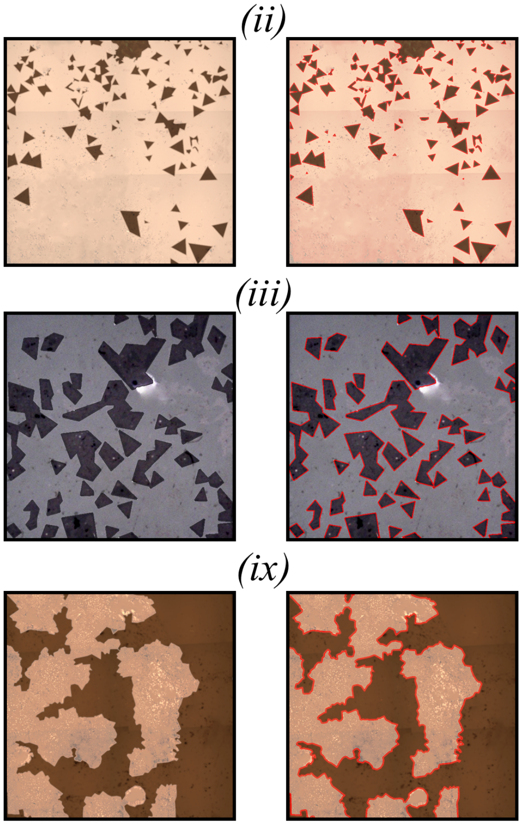

Figure S11: Optical microscopy of samples *(ii)*, *(iii)*, and *(ix)*. On the left side are the images used in Figures S9 and S10, on the right side the edges are highlight in red. All images have 1x1 mm^2.

S2 Rashba-Edelstein Efffect

There is an ongoing debate about the specific mechanism underlying the spin-to-charge conversion phenomenon in MoS_2. Concerning the 2D semiconductor states, experimental studies have proposed the Rashba-Edelstein effect as the main effect [6, 7, 8, 9, 10]. In contrast, theoretical works have attributed the origin of the spin-to-charge conversion to the orbital and valley Hall effects [11, 12], but there is no consensus yet. To get more insight concerning the microscopic origins of the spin-to-charge conversion in the MoS_2, one can notice from equation S4 that the spin pumping is expected to depend on the thickness of the non-magnetic layer. The thickness dependency relies on the spin-dependent scattering in the MoS_2. For the SHE, OHE, and VHE, this scattering is expected to occur along the path that the spin current enters the film, which is related to spin (or orbital) diffusion lengths. For these Hall effects, the spin pumping increases for small thicknesses (compared to the diffusion lengths) and saturates for thicker films [13, 14, 15, 16]. On the other hand, the inverse Rashba-Edelstein effect (IREE) results from a broken inversion symmetry. In the hexagonal MoS_2, it manifests under two conditions. Firstly, in a monolayer with a spatial group of P-6m2, noncentrosymmetry breaks the inversion symmetry. Secondly, in a multilayer with a spatial group of P63/mmc, although centrosymmetric, the layered structure allows the inversion symmetry to break at the interface. In both scenarios (mono- and multilayer), the IREE is expected only at the YIG/MoS_2 interface, rendering it independent of MoS_2 thickness.

To verify the spin pumping origin of the semiconductor states present in MoS_2 area states, the YIG samples were fully covered (whole area) with different thicknesses of MoS_2. For this, in addition to using CVD, which was used for the monolayer MoS_2 growth, we have used an automated exfoliated method to grow thicker films of MoS_2 directly to the YIG surface [17]. Fig. S12 shows the spin pumping, α_{SP}, as a function of the MoS_2 thickness. As can be seen, the spin pumping obtained from samples ranging from monolayer to bulk exhibits a thickness-independent behavior, which is in agreement with previously published data [17]. In conclusion, the observed effect is purely interfacial, agreeing with the behavior expected from the IREE.

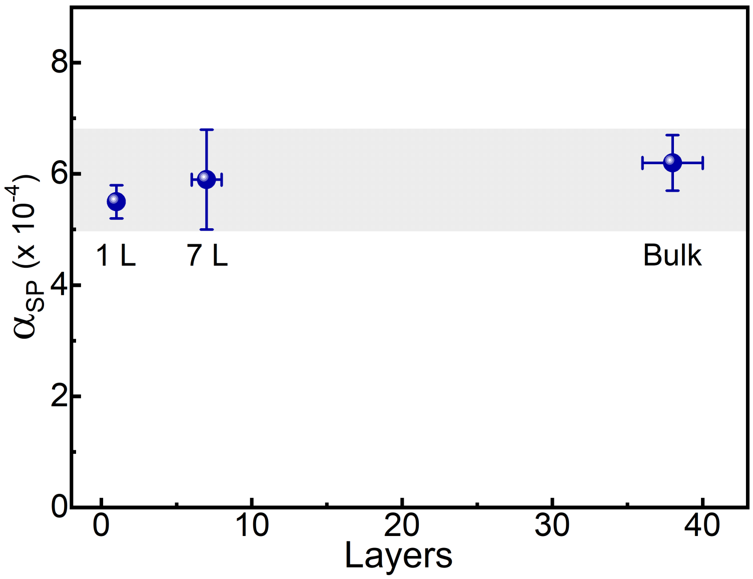

Figure S12: Dependence of spin pumping (α_{SP}) as a function of the number of MoS_2 layers. The uncertainty in α_{SP} was obtained from the data fit.

S3 Ferromagnetic resonance and spin pumping

Ferromagnetic resonance (FMR) is a powerful tool for investigating magnetic properties. Figure S13 shows an example of the FMR power absorption derivative measurement recorded at 10.0 GHz. The solid red symbols represent the data and the black line is a derivative Lorentzian function fit (for details see [17, 18, 19, 20]). One of the most important parameters obtained from this measurement is the FMR linewidth (ΔH), which is directly related to the magnetic Gilbert damping (α). Experimentally, it is common to measure the FMR at several frequencies (ω_{RF}) to obtain a more accurate Gilbert damping troght the equation:

$$\Delta H = \frac{\alpha}{|\gamma|}\omega_{RF} + \Delta H_0 \quad \text{(S1)}$$

where ΔH_0 denotes film inhomogeneity line broadening, and $|\gamma| = 2.802$ MHz Oe^{-1} is the electron gyromagnetic ratio.

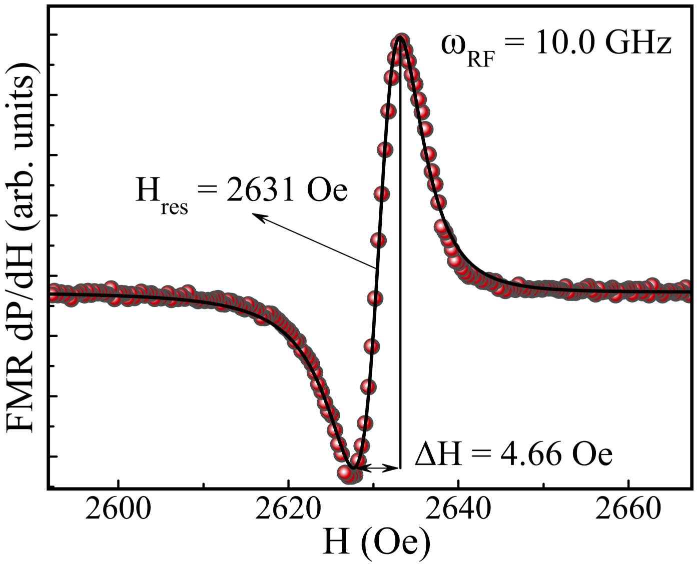

Figure S13: Exemple of a ferromagnetic resonance measurement recorded at 10.0 GHz. The red circles represents the FMR data and the black is the fit.

As described in the main manuscript, Gilbert damping was measured using two different ferromagnetic resonance (FMR) setups [17, 18], in both

cases using an AC magnetic field modulation of 0.5 Oe and 45 kHz for lock-in detection. In the first setup, a fixed frequency cavity configuration with a frequency of 9.8 GHz was used for the light-exited measurements. An example of the FMR measurement and the enhancement of ΔH and α due to spin pumping in sample S4 is shown in Fig. S14.

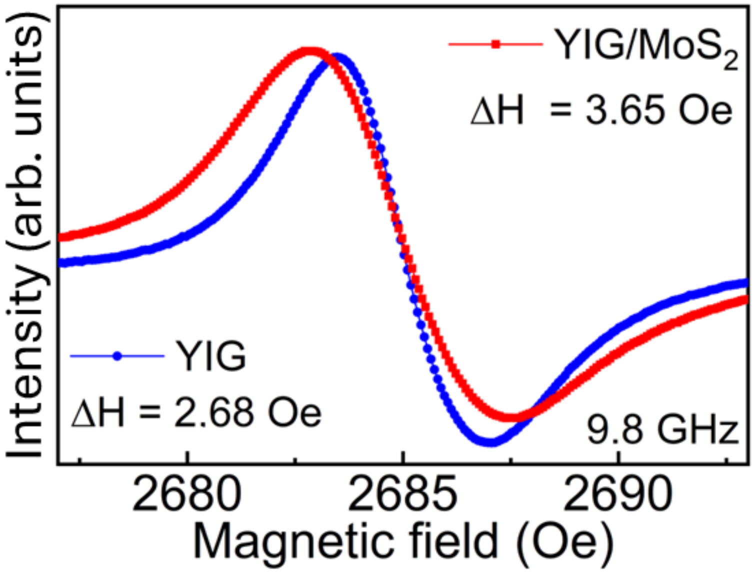

Figure S14: Ferromagnetic resonance measurement of sample S4 recorded in a fixed frequency cavity configuration at a frequency of 9.8 GHz. In blue the bare YIG and in red the YIG/MoS_2 heterostructure.

In the second setup, a broadband coplanar waveguide was used from 3 to 14 GHz for the FMR measurements without light incidence. The linear dependence of the FMR linewidth as a function of frequency fitted by the Equation S1 for samples S1 to S4 is shown in Fig. S15(a) to (d).

The spin pumping (α_{SP}) of each sample has been evaluated by the enhancement of the Gilbert damping of the YIG before and after the MoS_2 transfer and can be quantified by:

$$\alpha_{SP} = \alpha_{YIG/MoS_2} - \alpha_{YIG} \quad \text{(S2)}$$

where α_{YIG} is the Gilbert damping measured before the MoS_2 transfer and α_{YIG/MoS_2} after the transfer, as can be seen in Figure S15.

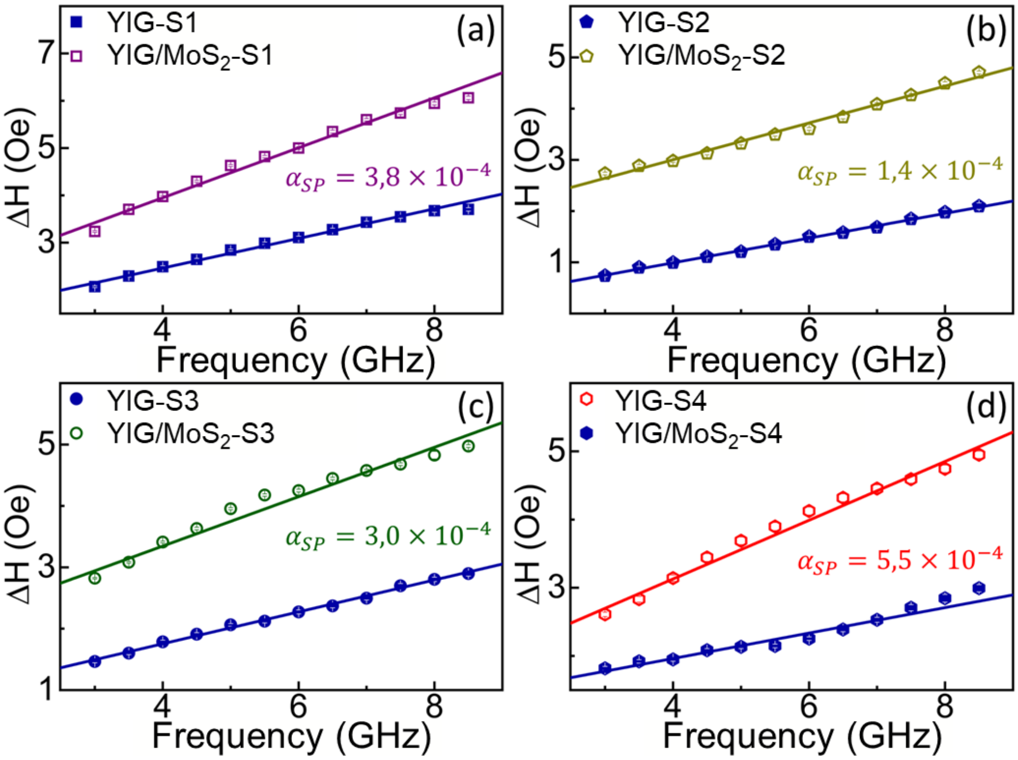

Figure S15: FMR linewidth as a function of frequency and the linear fit yields in Gilbert damping. Samples S1 to S4 are represented by the purple square (a), yellow pentagon (b), green circle (c), and red hexagon (d), respectively. The solid blue circles represent the bare YIG.

It is important to note that both Gilbert damping and spin pumping were obtained only by fitting the experimental data and using the electron gyromagnetic ratio, which can also be obtained from FMR measurements.

Another important parameter obtained from the FMR measurement is the resonance magnetic field (H_{res}), as shown in Figure S13. From the dependence of the resonance frequency on the resonance field, the effective magnetization and the effective gyromagnetic ratio can be obtained by fitting the Kittel equation:

$$\omega_{RF} = |\gamma_{\text{eff}}|\sqrt{H_{\text{res}}(H_{\text{res}} + 4\pi M_{\text{eff}})} \tag{S3}$$

where ω_{RF} is the radio frequency, $|\gamma_{\text{eff}}|$ is the effective gyromagnetic ratio,

H_{res} is the resonance magnetic field corresponding to the RF, and $4\pi M_{\mathrm{eff}}$ is the effective magnetization.

The effective magnetization and the effective gyromagnetic ratio obtained by fitting the Kittel equation for all the samples used in this work are shown in Table 1.

Table 1: Magnetic and spin pumping properties. Values of effective magnetization ($4\pi\mathrm{M}_{\mathrm{eff}}$), effective gyromagnetic ratio ($|\gamma_{\mathrm{eff}}|$) and effective spin mixing conductance ($g_{\uparrow\downarrow}$). The labels of the samples refer to the identification in Figure S8.

Sample	M_{eff} (G)	$\lvert\gamma_{\mathrm{eff}}\rvert$ (MHz/Oe)	$g_{\uparrow\downarrow} \times 10^{18}(\mathrm{m}^{-2})$
(i)	1839 ± 3	2.819 ± 0.001	2.1 ± 0.5
(ii)	1961 ± 3	2.819 ± 0.001	1.9 ± 0.3
(iii)	2040 ± 4	2.825 ± 0.001	2.1 ± 0.3
(iv)	2256 ± 7	2.818 ± 0.001	1.6 ± 0.4
(v)	1871 ± 7	2.790 ± 0.001	1.9 ± 0.6
(vi)	2073 ± 6	2.822 ± 0.001	1.7 ± 0.3
(vii)	1915 ± 8	2.789 ± 0.001	1.5 ± 0.7
(viii)	2310 ± 6	2.825 ± 0.001	1.2 ± 0.6
(ix)	2111 ± 5	2.819 ± 0.001	0.9 ± 0.3
(x)	1886 ± 8	2.795 ± 0.001	0.7 ± 0.5
(xi)	2148 ± 4	2.819 ± 0.001	0.5 ± 0.3
(xii)	2023 ± 3	2.826 ± 0.001	1.6 ± 0.4
(xiii)	2132 ± 2	2.811 ± 0.001	3.2 ± 0.5

Although spin pumping can be obtained through the enhancement of the Gilbert damping, which can be experimentally probed directly from the FMR measurements, sometimes it is essential to go deep into the theory of spin pumping to get more insight into the physical nature of the effects behind the promotion of the spin current injection in different types of materials. In the original work, which is still paving the theory of spin pumping, Tserkovniak [21, 22] proposes that the spin current injection in ferromagnetic/normal metal bilayer should follow the equation 1 in the main text. This equation was proposed in a context where the Spin Hall Effect (SHE) was the main, if not the only, effect responsible for spin pumping, which is no longer valid. Recently, several other mechanisms have been shown to promote spin current injections in bilayers, in particular the Rashba Edelstein Effect (REE), the

Valley Hall Effect (VHE), and the Orbital Hall Effect (OHE). Due to the physical nature of the effects, one might expect VHE and OHE to behave similarly to SHE. However, this is not the case for REE.

To clarify some points in the manuscript on the origin of the SP in YIG/MoS_2, we present here another form of Equation 1, which improves the clarity of the physical quantities involved in the SP process. First, the SP can be described as:

$$\begin{aligned}\alpha_{SP} &= \alpha_{FM/M} - \alpha_{FM} \\ &= \frac{g_L g^{\uparrow\downarrow}}{4\pi\mu}\left[1 + \frac{\sqrt{3}g^{\uparrow\downarrow}}{\sqrt{\varepsilon}Sk_F^2 tanh(\frac{L}{\lambda_{SD}})}\right]^{-1}\end{aligned} \tag{S4}$$

where α_{FM} is the Gilbert damping of the bare FM layer, $\alpha_{FM/NM}$ is the Gilbert damping of the FM coupled with the normal metal layer, ε is the spin flip probability at each scattering which is proportional to the spin-orbit coupling, S is the FM/NM interface area, g_L is the g factor, μ is the total film magnetic moment in units of μ_B, $g^{\uparrow\downarrow}$ is the interfacial mixing conductance, k_F is the Fermi wave vector, L is the YIG film thickness, and λ_{SD} is the spin diffusion length.

This equation already shows that the α_{SP} depends on the surface area (S) in the second term inside the brackets. To make the area dependence clearer, the equation S4 can be expanded with the interfacial conductance [23]:

$$g^{\uparrow\downarrow} \approx (Sk_F^2)/(4\pi) \tag{S5}$$

Substituting the interfacial mixing conductance given in the equation S5 into the spin pumping equation S4, one obtains:

$$\begin{aligned}\alpha_{SP} &= \frac{g_L \frac{Sk_F^2}{4\pi}}{4\pi\mu}\left[1 + \frac{\sqrt{3}\frac{Sk_F^2}{4\pi}}{\sqrt{\varepsilon}Sk_F^2 tanh(\frac{L}{\lambda_{SD}})}\right]^{-1} \\ &= \frac{g_L Sk_F^2}{(4\pi)^2\mu}\left[1 + \frac{\sqrt{3}Sk_F^2}{4\pi\sqrt{\varepsilon}Sk_F^2 tanh(\frac{L}{\lambda_{SD}})}\right]^{-1} \\ &\approx \frac{g_L k_F^2}{(4\pi)^2\mu}S\left[1 + \frac{\sqrt{3}}{4\pi\sqrt{\varepsilon}tanh(\frac{L}{\lambda_{SD}})}\right]^{-1}\end{aligned} \tag{S6}$$

which clearly shows that the spin pumping should scale linearly with the interface area for systems with spin, orbital and Valley Hall effects.

In addition, it is also possible to obtain the effective spin mixing conductance at the YIG/MoS_2 interface, see Table 1, which was calculated using the equation:

$$g_{\uparrow\downarrow} = \frac{4\pi M_{\text{eff}} t_{\text{f}} \hbar}{|\gamma_{\text{eff}}|} \alpha_{sp} \tag{S7}$$

where t_f is the ferromagnetic thickness, which is 50 nm for all samples, $4\pi M_{\text{eff}}$ and $|\gamma_{\text{eff}}|$ are the effective magnetization and effective gyromagnetic ratio from Table 1, and $\hbar$ is the reduced Planck constant.

The spin mixing conductance is commonly used to compare spin pumping in systems with different ferromagnetic materials as spin current injectors, primarily to account for variations in magnetic properties and thickness. As shown in Table 1, the effective magnetization values of the YIG samples in this study range from 1839 to 2310 G, which can lead to differences in between the α_{SP} and the $g_{\uparrow\downarrow}$. To visualize these differences and further validate the robustness of the V-shaped dependence of spin pumping on the A_{Total}/P_{Total} ratio (Figure 1 (e)), we present the spin mixing conductance as a function of the A_{Total}/P_{Total} ratio in Figure S16. As seen, the same trend is observed in both cases. In fact, the dispersion of the data points relative to the linear fit is even smaller for the spin mixing conductance compared to α_{SP}.

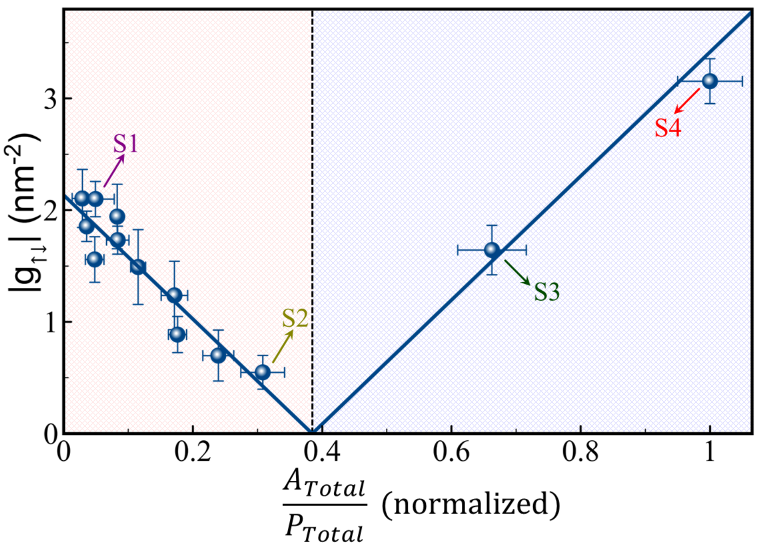

Figure S16: Spin mixing conductance ($g_{\uparrow\downarrow}$) dependence as a function of the total MoS_2 area divided by the total perimeter (A_{Total}/P_{Total}). The uncertainty in α_{SP} was obtained from the data fit and in area and perimeter from the image analysis.

S4 MoS_2 monolayer bandgap

It is well known that the MoS_2 monolayer has a direct band gap in the K-point of the Brillouin zone and the optical bandgap is between 1.8 and 1.9 eV, therefore resonating in the absorption of a red-light laser with a wavelength of about 633 nm and thus giving the A exciton peak in the photoluminescence. However, it is important to note that there is a difference between the optical bandgap and the electronic bandgap. The electronic bandgap (E_g) is characterized by a single particle or quasiparticle excitation and is defined by the sum of the energies to separately tunnel an electron and a hole. In other words, it is the energy required to move an electron from the valence band to the conduction band. The optical band gap (E_{opt}), on the other hand, describes the energy required to create an exciton (a correlated two-particle electron-hole pair) by optical absorption. The difference between these energies (E_g - E_{opt}) gives the exciton binding energy (E_b) [24].

In conventional semiconductor materials such as Si, Ge, GaAs, etc. the exciton binding energies are in the order of a few meV and are therefore sometimes neglected [25, 26]. On the other hand, the 1H TMDs, such as the MoS_2 monolayer, present several properties (weak dielectric screening, strong geometrical confinement, strong Coulomb interaction, etc.), which together lead to several different types of exciton formation and also to binding energies in the order of 0.5 eV, which cannot be neglected [27]. Therefore, photoluminescence is used to determine the optical bandgap rather than the electronic bandgap and is not an accurate representation of the electronic bandgap. In fact, until recently there was a lack of data to directly probe the electronic bandgap of TMDs, which is no longer the case [24, 27, 28, 29].

Part of this confusion arises from the coincidence of the experimental values of the optical bandgap through photoluminescence and the calculated values obtained by Density Functional Theory (DFT). However, it is well known that conventional DFT strongly underestimates the band gaps, usually due to the local density or gradient approximations - for example, the gap estimated by DFT for bulk MoS_2 is typically 0.76 eV, which is significantly smaller than the experimental values, which are of the order of 1.3 eV. To obtain a more accurate estimate of the value of the band gap, a quasiparticle approach in the GW approximation [30] is often used - with this approach the gap for bulk MoS_2 is found to be 1.3 eV, in agreement with the experimental value.

For the case of the MoS_2 monolayer, the values for the electronic gap

using the GW method were found to be in the range of 2.76 eV, which is 0.9 eV higher than the typical photoluminescence experimental results. However, this difference is explained by the high binding energy of the inherent 2D screening of the Coulomb interaction in the monolayer. Therefore, better estimates of the electronic band gap of the MoS_2 monolayers should be provided by the GW approximations, which yield a value of 2.76 eV, and/or electronic measurements, which found an electronic band gap of 2.4 eV for the monolayer, and also considering that the substrate can also influence in the exact values of the band gap [31, 32, 33, 34].

S5 FMR before and after light

FMR measurements were performed under light incidence using three laser diodes with different wavelengths. The lasers used have a power of up to 50 mW and may have a small component of circular polarization. In order to ensure that the illumination does not alter the properties of YIG/MoS_2, especially the magnetic ones, we have adopted a protocol to measure the ferromagnetic resonance of all samples before any light illumination and to compare it with the ferromagnetic resonance of the samples after the exposures, measured without illumination. In Fig. S17, we showed the field dependence of the derivative of the radiofrequency absorption (FMR measurements) at 9.8 GHz for samples S1 (a), S2 (b), S3 (c) and S4 (d). The solid black squares represent the measurements before any light incidence. The measurements without light incidence but after the light incidence of samples S1 to S4 are represented by the purple square (a), yellow pentagon (b), green circle (c), and red hexagon (d), respectively. As can be seen, the measurements of the same sample before and after exposure to light are in perfect agreement. This shows that the light has not damaged the MoS_2 layer, and that the experimental procedure is fully reversible. In addition, the light-induced spin pumping measurements were not performed simply by increasing or decreasing the light intensity. The data shown in Figure 3 were acquired in a non-sequential order of light intensity. This non-sequential data acquisition protocol further confirms the absence of light damage to the samples and the reversibility of the process.

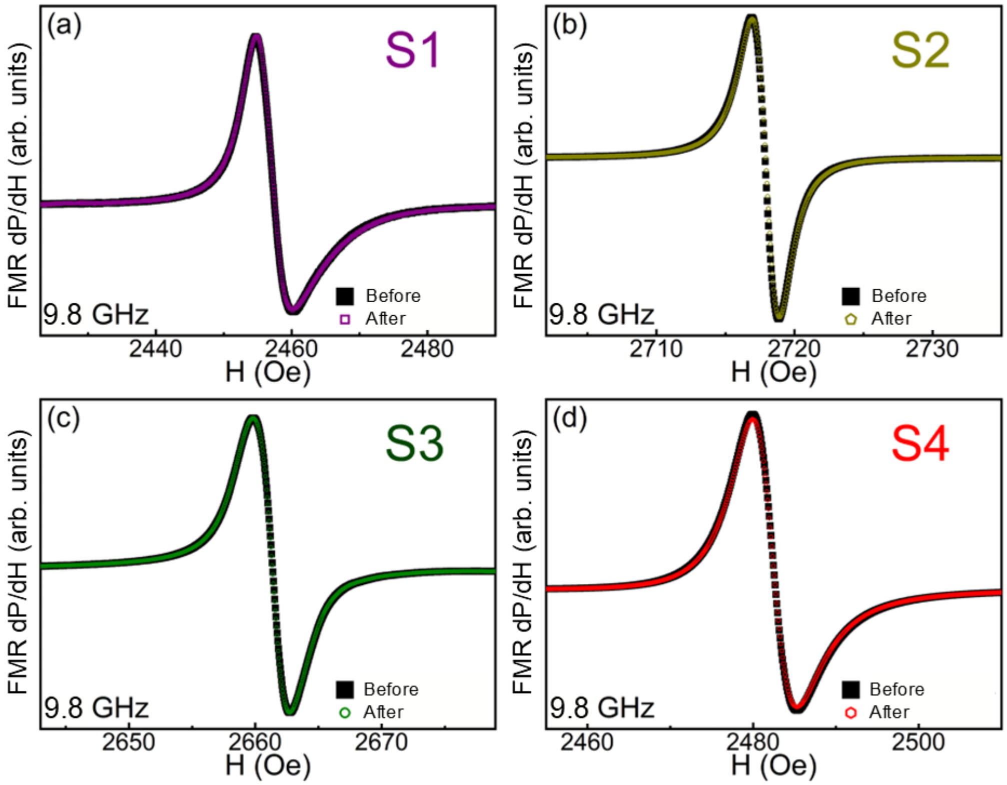

Figure S17: FMR measurements of samples S1 to S4 before (solid black squares) and after light irradiation; S1 purple square (a), S2 yellow pentagon (b), S3 green circle (c) and S4 red hexagon (d), respectively.

References

[1] Syed Hamza Safeer et al. "CVD growth and optical characterization of homo and heterobilayer TMDs". In: *Journal of Applied Physics* 132.2 (2022), p. 024301.

[2] Syed Hamza Safeer et al. "Sodium-Mediated Low-Temperature Synthesis of Monolayers of Molybdenum Disulfide for Nanoscale Optoelectronic Devices". In: *ACS Appl. Nano Mater.* 4.4 (2021), pp. 4172–4180. ISSN: 25740970. DOI: 10.1021/acsanm.1c00491.

[3] Syed Hamza Safeer et al. "Etching-Free Transfer and Nanoimaging of CVD-Grown MoS2 Monolayers". In: *The Journal of Physical Chemistry C* 125.38 (2021), pp. 21011–21017.

[4] Yi-Hsien Lee et al. "Synthesis of large-area MoS2 atomic layers with chemical vapor deposition". In: *Advanced materials* 24.17 (2012), pp. 2320–2325.

[5] Caroline A Schneider, Wayne S Rasband, and Kevin W Eliceiri. "NIH Image to ImageJ: 25 years of image analysis". In: *Nature methods* 9.7 (2012), pp. 671–675.

[6] JBS Mendes et al. "Efficient spin to charge current conversion in the 2D semiconductor MoS2 by spin pumping from yttrium iron garnet". In: *Applied Physics Letters* 112.24 (2018), p. 242407.

[7] Himanshu Bangar et al. "Large Spin-To-Charge Conversion at the Two-Dimensional Interface of Transition-Metal Dichalcogenides and Permalloy". In: *ACS Applied Materials & Interfaces* 14.36 (2022), pp. 41598–41604.

[8] Wei Zhang et al. "Research Update: Spin transfer torques in permalloy on monolayer MoS2". In: *APL Materials* 4.3 (2016), p. 032302.

[9] Qiming Shao et al. "Strong Rashba-Edelstein effect-induced spin–orbit torques in monolayer transition metal dichalcogenide/ferromagnet bilayers". In: *Nano letters* 16.12 (2016), pp. 7514–7520.

[10] Cheng Cheng et al. "Spin to charge conversion in MoS _{2} monolayer with spin pumping". In: *arXiv preprint arXiv:1510.03451* (2015).

[11] Tarik P Cysne et al. "Disentangling orbital and valley Hall effects in bilayers of transition metal dichalcogenides". In: *Physical review letters* 126.5 (2021), p. 056601.

[12] Luis M Canonico et al. “Orbital Hall insulating phase in transition metal dichalcogenide monolayers”. In: *Physical Review B* 101.16 (2020), p. 161409.

[13] Carl T Boone et al. “Spin transport parameters in metallic multilayers determined by ferromagnetic resonance measurements of spin-pumping”. In: *Journal of Applied Physics* 113.15 (2013).

[14] Carl T Boone et al. “Spin-scattering rates in metallic thin films measured by ferromagnetic resonance damping enhanced by spin-pumping”. In: *Journal of Applied Physics* 117.22 (2015).

[15] M Caminale et al. “Spin pumping damping and magnetic proximity effect in Pd and Pt spin-sink layers”. In: *Physical Review B* 94.1 (2016), p. 014414.

[16] Hiroyasu Nakayama et al. “Geometry dependence on inverse spin Hall effect induced by spin pumping in Ni 81 Fe 19/Pt films”. In: *Physical Review B* 85.14 (2012), p. 144408.

[17] Rodrigo Torrão Victor et al. “Automated mechanical exfoliation technique: a spin pumping study in YIG/TMD heterostructures”. In: *Nanoscale Horizons* 8.11 (2023), pp. 1568–1576.

[18] R Torrão et al. “Reproducible low Gilbert damping yttrium iron garnet by magnetron sputtering”. In: *Journal of Alloys and Compounds* 923 (2022), p. 166300.

[19] AM Gonçalves et al. “Spin torque ferromagnetic resonance with magnetic field modulation”. In: *Applied Physics Letters* 103.17 (2013), p. 172406.

[20] AM Gonçalves et al. “Oscillatory interlayer coupling in spin Hall systems”. In: *Scientific reports* 8.1 (2018), pp. 1–6.

[21] Yaroslav Tserkovnyak, Arne Brataas, and Gerrit EW Bauer. “Spin pumping and magnetization dynamics in metallic multilayers”. In: *Physical Review B* 66.22 (2002), p. 224403.

[22] Gerrit EW Bauer et al. “Universal angular magnetoresistance and spin torque in ferromagnetic/normal metal hybrids”. In: *Physical Review B* 67.9 (2003), p. 094421.

[23] K Xia et al. “Spin torques in ferromagnetic/normal-metal structures”. In: *Physical Review B* 65.22 (2002), p. 220401.

[24] Miguel M Ugeda et al. "Giant bandgap renormalization and excitonic effects in a monolayer transition metal dichalcogenide semiconductor". In: *Nature materials* 13.12 (2014), pp. 1091–1095.

[25] Ronald L Greene, Krishan K Bajaj, and Dwight E Phelps. "Energy levels of Wannier excitons in G a A s- Ga 1- x Al x As quantum-well structures". In: *Physical Review B* 29.4 (1984), p. 1807.

[26] RC Miller et al. "Observation of the excited level of excitons in GaAs quantum wells". In: *Physical Review B* 24.2 (1981), p. 1134.

[27] Thomas Mueller and Ermin Malic. "Exciton physics and device application of two-dimensional transition metal dichalcogenide semiconductors". In: *npj 2D Materials and Applications* 2.1 (2018), p. 29.

[28] Keliang He et al. "Tightly bound excitons in monolayer WSe 2". In: *Physical review letters* 113.2 (2014), p. 026803.

[29] Alexey Chernikov et al. "Exciton binding energy and nonhydrogenic Rydberg series in monolayer WS 2". In: *Physical review letters* 113.7 (2014), p. 076802.

[30] Lars Hedin. "New method for calculating the one-particle Green's function with application to the electron-gas problem". In: *Physical Review* 139.3A (1965), A796.

[31] Yu Li Huang et al. "Bandgap tunability at single-layer molybdenum disulphide grain boundaries". In: *Nature communications* 6.1 (2015), p. 6298.

[32] Ivan N Yakovkin. "Dirac cones in graphene, interlayer interaction in layered materials, and the band gap in MoS2". In: *Crystals* 6.11 (2016), p. 143.

[33] Junga Ryou et al. "Monolayer MoS2 bandgap modulation by dielectric environments and tunable bandgap transistors". In: *Scientific reports* 6.1 (2016), p. 29184.

[34] Rafael Roldán et al. "Electronic properties of single-layer and multilayer transition metal dichalcogenides MX2 (M= Mo, W and X= S, Se)". In: *Annalen der Physik* 526.9-10 (2014), pp. 347–357.
